# Amino acids inhibit kynurenic acid formation via suppression of kynurenine uptake or kynurenic acid synthesis in rat brain *in vitro*

**DOI:** 10.1186/s40064-015-0826-9

**Published:** 2015-02-01

**Authors:** Airi Sekine, Misaki Okamoto, Yuka Kanatani, Mitsue Sano, Katsumi Shibata, Tsutomu Fukuwatari

**Affiliations:** Department of Nutrition, School of Human Cultures, The University of Shiga Prefecture, 2500 Hassaka, Hikone, Shiga 522-8533 Japan

**Keywords:** Amino acids, Kynurenic acid, Kynurenine, α7 nicotinic acetylcholine receptor, Dopamine, Neuropsychiatric disorders

## Abstract

The tryptophan metabolite, kynurenic acid (KYNA), is a preferential antagonist of the α7 nicotinic acetylcholine receptor at endogenous brain concentrations. Recent studies have suggested that increase of brain KYNA levels is involved in psychiatric disorders such as schizophrenia and depression. KYNA-producing enzymes have broad substrate specificity for amino acids, and brain uptake of kynurenine (KYN), the immediate precursor of KYNA, is via large neutral amino acid transporters (LAT). In the present study, to find out amino acids with the potential to suppress KYNA production, we comprehensively investigated the effects of proteinogenic amino acids on KYNA formation and KYN uptake in rat brain *in vitro.* Cortical slices of rat brain were incubated for 2 h in Krebs-Ringer buffer containing a physiological concentration of KYN with individual amino acids. Ten out of 19 amino acids (specifically, leucine, isoleucine, phenylalanine, methionine, tyrosine, alanine, cysteine, glutamine, glutamate, and aspartate) significantly reduced KYNA formation at 1 mmol/L. These amino acids showed inhibitory effects in a dose-dependent manner, and partially inhibited KYNA production at physiological concentrations. Leucine, isoleucine, methionine, phenylalanine, and tyrosine, all LAT substrates, also reduced tissue KYN concentrations in a dose-dependent manner, with their inhibitory rates for KYN uptake significantly correlated with KYNA formation. These results suggest that five LAT substrates inhibit KYNA formation via blockade of KYN transport, while the other amino acids act via blockade of the KYNA synthesis reaction in brain. Amino acids can be a good tool to modulate brain function by manipulation of KYNA formation in the brain. This approach may be useful in the treatment and prevention of neurological and psychiatric diseases associated with increased KYNA levels.

## Background

Tryptophan was mainly metabolized through the kynurenine (KYN) pathway in the mammalian brain. Kynurenic acid (KYNA), a product of this pathway, is a negative allosteric modulator of the α7 nicotinic acetylcholine receptor at endogenous concentration, and a competitive antagonist of glycine co-agonist sites of the *N*-methyl-D-aspartic acid receptor (Kessler et al. 1989; Hilmas et al. 2001; Schwarcz and Pellicciari 2002). In particular, nanomolar increases in KYNA reduces dopaminergic and glutamatergic neurotransmission (Carpenedo et al. 2001; Rassoulpour et al. 2005), and contributes to cognitive dysfunction (Erhardt et al. 2004; Chess and Bucci 2006; Chess et al. 2007; Chess et al. 2009). While decreases in endogenous KYNA augment dopaminergic, acetylcholinergic and glutamatergic neurotransmission (Amori et al. 2009a; Zmarowski et al. 2009; Konradsson-Geuken et al. 2010), and lead to enhanced cognitive abilities (Potter et al. 2010; Kozak et al. 2014). In humans, patients with schizophrenia show higher KYNA levels in the prefrontal cortex and cerebrospinal fluid (Erhardt et al. 2001; Schwarcz et al. 2001; Linderholm et al. 2010). Based on these findings, it has been suggested that KYNA is involved in the pathophysiology of psychiatric disorders including schizophrenia (Erhardt et al. 2007; Erhardt et al. 2009), and thus, suppression of KYNA production may contribute to prevention or improvement in these disorders.

Astrocytes uptake KYN, the immediate bioprecursor of KYNA, from blood stream, and KYN is metabolized to KYNA. Two factors regulate KYNA production in the brain: kynurenine amino transferase (KAT) activity and availability of KYN (Turski et al. 1989). Four KATs have been identified in the mammalian brain, these KATs have broad substrate specificity for amino acids, and several amino acids competitively inhibit KATs for KYNA production (Okuno et al. 1991; Guidetti al. 2007; Han et al. 2010). Hence, amino acids may suppress KYNA production via KAT inhibition in the brain. Astrocytes uptake peripheral KYN from blood stream via large neutral amino acid transporters (LATs). LATs are known to transport both branched chain amino acids (e.g., valine, leucine and isoleucine) and aromatic amino acids (e.g., tyrosine, phenylalanine, and tryptophan). Several findings show that LATs transport amino acids with higher affinity than KYN in tumor cell lines (Fukui et al. 1991; Speciale et al. 1989; Asai et al. 2008; Yanagida et al. 2001), and changes in physiological concentrations of these amino acids may affect KYN transport into the brain.

In the present study, we comprehensively investigated the effects of amino acids on suppression of KYNA production via inhibition of KYN uptake and KYNA synthesis in the brain. We used brain slices to determine the inhibitory effects on KYNA synthesis, KYN uptake and KYNA synthesis at physiological KYN concentrations. Our findings demonstrate that amino acids are a good tool for modulating brain function by manipulating KYNA formation in the brain.

## Methods

### Animals

Male Wistar rats (7–10 weeks old) were obtained from CLEA Japan (Tokyo, Japan). Rats were allowed free access to food and water. The animal room was maintained at a temperature of 22°C with 60% humidity and a 12-h light/12-h dark cycle (light onset at 6:00 a.m.). Care and treatment of experimental animals conformed to the University of Shiga Prefecture guidelines for ethical treatment of laboratory animals (reference number: 24-9).

### Chemicals

L-Kynurenine sulfate salt, KYNA, and L-tyrosine disodium salt hydrate were purchased from Sigma Chemical Co. (St. Louis, MO, USA). L-Alanine, L-arginine, L-asparagine, L-aspartate, L-cysteine hydrochloride monohydrate, L-glutamine, L-glutamate, L-glycine, L-histidine, L-isoleucine, L-leucine hydrochloride, L-lysine, L-methionine, L-phenylalanine, L-proline, L-serine, L-threonine, and L-valine were purchased from Wako Pure Chemical Industries (Osaka, Japan). Rodent diet (MF) was obtained from Oriental Yeast Co., Ltd (Tokyo, Japan). All other chemicals were of the highest commercially available purity.

### Tissue preparation

*De novo* formation of KYNA was established using tissue slices, as described previously Turski et al. (1989). Animals were killed by decapitation and brains removed rapidly. The cortex was rapidly dissected out and kept in a minimal volume of ice-cold Krebs-Ringer buffer (KRB: 118.5 mmol/L NaCl, 4.8 mmol/L KCl, 1.8 mmol/L CaCl_2_, 1.2 mmol/L MgSO_4_, 16.2 mmol/L NaH_2_PO_4_, 5.0 mmol/L glucose, pH 7.4). Tissue slices (1 × 1 mm) were made using a McIlwain tissue slicer (Muromachi Kikai Co., Ltd, Tokyo, Japan), and placed in ice-cold KRB until the start of experiments (<1 h).

### *In vitro* screening of amino acids regulating *de novo* KYNA formation

Routinely, seven tissue slices were placed in each culture well (seven slices per well, ~1 mg of total protein) containing a final volume of 1 mL ice-cold KPB and final concentration of 1 mmol/L each amino acid for screening, or 3–3000 mmol/L each amino acid for dose–response assays. After 10 min pre-incubation at 37°C in an oxygenated shaking water bath, a final physiological concentration of 2 μmol/L KYN was added to each well. After 2 h incubation at 37°C, plates were placed on ice. Because assessment of the time course of KYNA concentration in KRB showed linear increases up to 4 h of incubation (Turski et al. 1989), biochemical viability of brain tissue was maintained during the study period. The medium was rapidly separated from the tissue and acidified with 100 μL of 1 mol/L HCl for subsequent KYNA measurements. We described KYNA concentration in the incubation medium as KYNA production, because more than 90% of newly synthesized KYNA readily liberate from tissue slices into the medium (Turski et al. 1989). The tissue slices were rapidly washed three times with 500 μL of KRB and sonicated using an ultrasonic cell breaker (Powersonic model 50; Yamato Kagaku, Tokyo, Japan) in 250 μL distilled water. The 200-μL aliquot of tissue slice suspension was acidified using 50 μL of 6% perchloric acid. After centrifugation (10 min, 12,000 × *g*, 4°C), the supernatant aliquot was used for KYN determination of component compound. A 50-μL aliquot of tissue slice suspension was used for protein determination using the Bradford assay (Bradford 1976).

### KYNA and KYN determination

KYNA concentration in the samples was determined by high-performance liquid chromatography with fluorescence detection (RF-20Axis; Shimadzu, Kyoto, Japan) at 344 nm excitation and 398 nm emission wavelengths (Shibata 1988). KYN concentration was determined by high-performance liquid chromatography with ultraviolet detection (SPD-10AV; Shimadzu) at a 365 nm wavelength (Holmes 1988).

### Statistical analysis

All data were expressed as mean ± SE. One-way analysis of variance with Dunnett’s Multiple Comparison Test was used for more than three-group comparisons. Sigmoid curves were generated by nonlinear regression analysis. We calculated the half-maximal inhibitory concentration (IC_50_ values in μmol/L) of each amino acid for KYNA production and KYN uptake using the equation “log (inhibitor) vs. response” by GraphPad Prism 5.0 (GraphPad Software, San Diego, CA, USA).

Correlation between inhibitory rates for KYNA production and tissue KYN concentration by amino acids were represented by linear regression of data. Inhibitory rates for KYNA production and tissue KYN concentration at each additional amino acid concentrations were described by the percentage of control values. Correlation between KYNA production and KYN uptake at various KYN concentrations in the KRB were also represented by linear regression of data. KYNA production and KYN uptake were described by the percentage of 2 μmol/L KYN control values. Pearson correlation coefficient was calculated respectively.

A *p*-value < 0.05 was considered significant. GraphPad Prism 5.0 (GraphPad Software, San Diego, CA, USA) was used for all analyses.

## Results

### Screening amino acids for suppression of KYNA production

**Figure 1:**
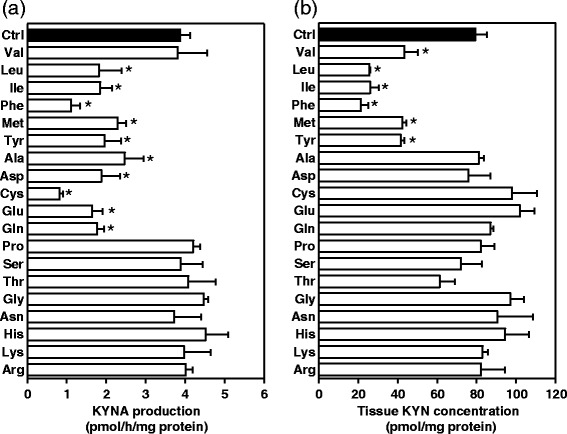
**Effect of 1 mmol/L amino acids on (a) KYNA production and (b) tissue KYN concentration in tissue slices from the cerebral cortex.** Experiments were performed as described in the text using 2 μmol/L KYN. KYNA and KYN were measured in incubation medium and tissue slice suspension, respectively. Values are expressed as mean ± SE (*n* = 3–7). *p < 0.05 versus control (Ctrl), determined by one-way analysis of variance with Dunnett’s multiple comparisons test.

To determine which amino acids suppress KYNA production *in vitro*, we comprehensively assessed 19 proteinogenic amino acids at 1 mmol/L using tissue slices from the rat cerebral cortex. Because commercially available tryptophan contains tryptophan metabolites (including KYN and KYNA), we excluded tryptophan from our initial list of 20 proteinogenic amino acids. The amount of KYNA in the extracellular medium was reduced by 40–60% by eight amino acids (leucine, isoleucine, methionine, alanine, tyrosine, glutamine, glutamate, and aspartate), and to approximately 25% by phenylalanine and cysteine (Figure 1a). Leucine, isoleucine, methionine, phenylalanine, and tyrosine also reduced tissue KYN concentrations to < 50% (Figure 1b). No significant difference was observed for tissue KYN concentrations using alanine, cysteine, glutamine, glutamate, and aspartate, which all reduced KYNA production. Although valine reduced tissue KYN concentrations to 50%, it did not suppress KYNA production

### Amino acid dose-dependent inhibition of KYNA production and KYN uptake

**Figure 2:**
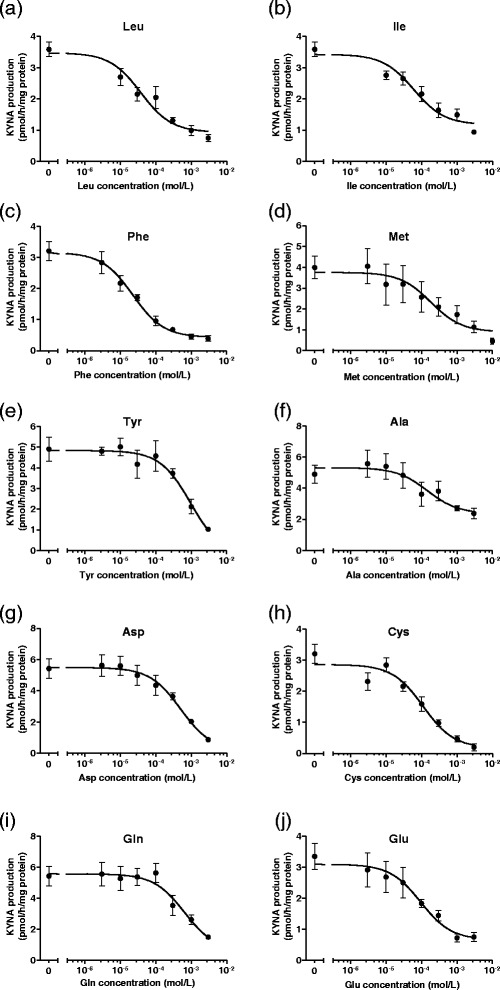
**Dose-dependent inhibition of KYNA production in tissue slices from the cerebral cortex by (a) leucine (Leu), (b) isoleucine (Ile), (c) phenylalanine (Phe), (d) tyrosine (Tyr), (e) methionine (Met), (f) cysteine (Cys), (g) aspartate (Asp), (h) glutamine (Gln), (i) alanine (Ala), and (j) glutamate (Glu).** Experiments were performed as described in the text using 2 μmol/L KYN. KYNA was measured in incubation medium. Values are expressed as mean ± SE (*n* = 4–6). Sigmoid curves were generated by nonlinear regression analysis using Graph Pad Prism 5.0.

**Figure 3:**
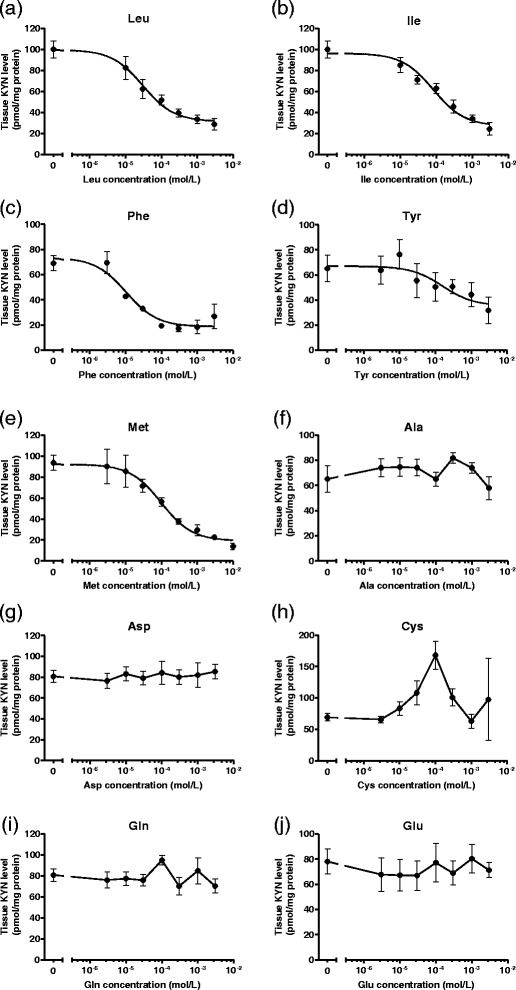
**Dose-dependent inhibition of tissue KYN concentration in tissue slices from cerebral cortex by (a) leucine (Leu), (b) isoleucine (Ile), (c) phenylalanine (Phe), (d) tyrosine (Tyr), (e) methionine (Met), (f) cysteine (Cys), (g) aspartate (Asp), (h) glutamine (Gln), (i) alanine (Ala), and (j) glutamate (Glu).** Experiments were performed as described in the text using 2 μmol/L KYN. KYN was measured in tissue slice suspension. Values are expressed as mean ± SE (*n* = 4–6). Sigmoid curves were generated by nonlinear regression analysis using Graph Pad Prism 5.0.

Since 10 of the 19 proteinogenic amino acids significantly reduced KYNA formation at 1 mmol/L, these amino acids were chosen for further investigation. To determine the precise capabilities of 10 amino acids to suppress KYNA production, each amino acid was added to KRB at concentrations varying from 3 μmol/L to 3 mmol/L. All 10 amino acids reduced KYNA production in a dose-responsive manner (Figure 2). Five amino acids (leucine, isoleucine, phenylalanine, methionine, and tyrosine) also reduced tissue KYN concentration in a dose-responsive manner (Figure 3). The other amino acids (alanine, aspartate, cysteine, glutamine, and glutamate) did not affect tissue KYN concentrations. In addition, we determined IC_50_ values for KYNA production and KYN uptake. The rank order of IC_50_ values for KYNA production was phenylalanine < leucine < isoleucine < glutamate < cysteine < alanine < methionine < aspartate < glutamine < tyrosine (Table 1), and for KYN uptake was phenylalanine > leucine > isoleucine > methionine > tyrosine (Table 1).

**Table 1:** **IC**_**50**_ **values for KYNA production and KYN uptake using 10 selected amino acids**

**Amino acids**	**IC**_**50**_ **for KYNA production (μmol/L)**	**IC**_**50**_ **for KYN uptake (μmol/L)**
Leucine	36.9	30.4
Isoleucine	60.1	83.6
Phenylalanine	22.5	10.4
Methionine	184	98.6
Tyrosine	970	159
Cysteine	110	—
Glutamate	94.9	—
Alanine	146	—
Aspartate	502	—
Glutamine	647	—

### Amino acid contribution of KYN uptake inhibition to KYNA production

**Figure 4:**
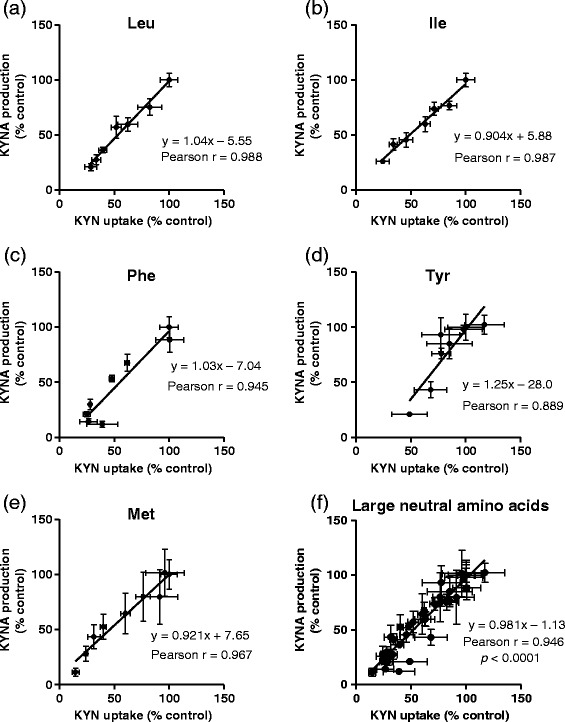
**Correlation between inhibitory rates for KYNA production and tissue KYN concentration by (a) leucine (Leu), (b) isoleucine (Ile), (c) phenylalanine (Phe), (d) tyrosine (Tyr), (e) methionine (Met), and (f) all five large neutral amino acids.** Values are expressed as mean ± SE (*n* = 4–6). Lines represent linear regression of data.

**Figure 5:**
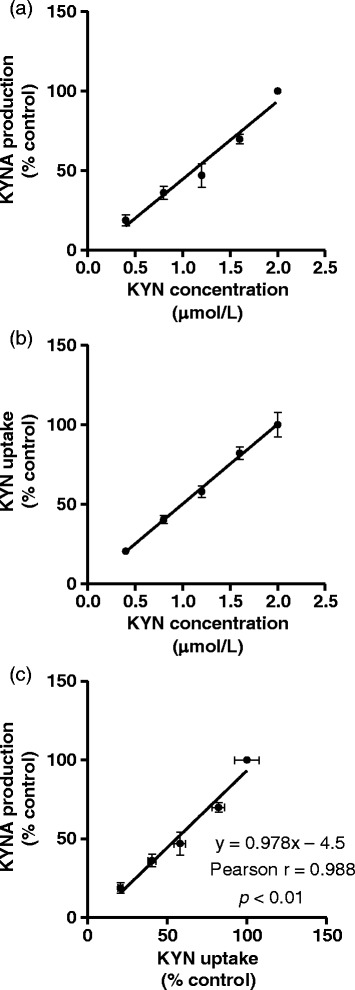
**Effect of KYN on (a) KYNA production and (b) KYN uptake in tissue slices from the cerebral cortex, and (c) correlation between KYNA production and KYN uptake.** Values are expressed as mean ± SE (*n* = 3). Lines represent linear regression of data.

To determine how inhibition of KYN uptake affects KYNA production, we selected five amino acids which reduced not only KYNA production but tissue KYN concentration, and examined the relationship between KYN uptake and KYNA production. Inhibitory rates of KYN uptake were significantly correlated with KYNA production by leucine (y = 1.04x – 5.6, *r* = 0.988; *p* < 0.0001), isoleucine (y = 0.904x + 5.9, *r* = 0.987; *p* < 0.0001), phenylalanine (y = 1.03x – 7.0, *r* = 0.945; *p* < 0.001), methionine (y = 1.25x – 28.0, *r* = 0.889; *p* < 0.01), and tyrosine (y = 0.921x – 7.7, *r* = 0.967; *p* < 0.0001) (Figure 4). Combining data from the respective amino acids showed significantly high correlation (y = 0.981x – 1.1, *r* = 0.946; *p* < 0.0001) (Figure 4f). To determine the direct relationship between tissue KYN concentration and KYNA production, cortical slices were incubated in KRB containing 0.4–2 μmol/L KYN. KYNA production and tissue KYN level were described the percentage of 2 μmol/L KYN control. With increasing KYN concentrations, KYNA production and tissue KYN concentration linearly increased in a dose-dependent manner (Figure 5a and b). Moreover, KYNA production was strongly correlated with tissue KYN concentration (y = 0.978x – 4.5, *r* = 0.985; *p* < 0.01) (Figure 5c). This relationship (including slope of the regression line) was the same for inhibitory effects of all five amino acids. These results suggest that inhibition of KYN uptake, but not KAT activity, contributes to inhibitory effects of these five large neutral amino acids on KYNA production.

## Discussion

Previous reports have shown that amino acids have the potential to suppress KYNA production via inhibition of KYN uptake and KYNA synthesis in the brain, therefore we comprehensively investigated the effects of proteinogenic amino acids on regulating KYNA production in rat brain *in vitro*. We show that 10 of 19 amino acids (specifically, leucine, isoleucine, phenylalanine, methionine, tyrosine, alanine, cysteine, glutamine, glutamate, and aspartate) significantly reduce KYNA production at the tissue level. Five (leucine, isoleucine, phenylalanine, methionine, and tyrosine) of these 10 amino acids also reduce tissue KYN concentration, with inhibition of KYNA production reflecting these reductions in KYN uptake. Our results suggest that these five amino acids suppress KYNA production via blockade of KYN transport, while the other five amino acids (alanine, cysteine, glutamine, glutamate, and aspartate) act via blockade of KYNA synthesis in the brain.

KYN is transported into the brain via LATs, which are Na^+^-independent neutral amino acids transporters. There are two LATs, LAT 1 and LAT 2, with the affinity of LAT 1 to large neutral amino acids higher than that of LAT 2. LAT 1 exhibits high-affinity transport of large neutral amino acids, including branched chain and aromatic amino acids, while LAT 2 has broader substrate specificities (Kanai et al. 1998; Segawa et al. 1999). The *K*_m_ value of LATs for KYN is ~160 μmol/L, 80 times higher than plasma KYN concentrations (Fukui et al. 1991; Speciale et al. 1989). In the present study, the amino acids that inhibited KYN uptake are consistent with substrate amino acids of LAT 1 rather than LAT 2, suggesting a critical role of LAT 1 in KYN uptake in the brain. *K*_m_ values of LAT 1 for leucine, isoleucine, methionine, phenylalanine, and tyrosine are 15–30 μmol/L, around physiological concentrations (Asai et al. 2008), indicating higher affinity than for KYN (Yanagida et al. 2001). The other amino acids (glutamine and aspartate) have low affinity for LAT 1 (*K*_m_ = 1.5–2 mmol/L), and are not substrates of LAT nor affect tissue KYN concentration. Histidine is also known to be a high affinity substrate for LAT 1 (*K*_m_ = 12.7 μmol/L), but did not reduce tissue KYN concentration in the present study. KYN is transported through either Na-independent or Na-dependent matter in tissue slice culture (Turski et al. Turski et al. 1989). In this study, only Na-independent LATs substrates such as leucine, but not Na-dependent LATs substrates such as glutamine, reduced KYN uptake. Na-independent transport rather than Na-dependent transport may contribute KYN uptake, and regulating of Na-independent LATs may be effective in modulating KYN uptake.

The mammalian brain expresses four KATs, KAT I (glutamine transaminase K, GTK; EC 2.6.1.64), KAT II (2-aminoadipate aminotransferase, ADA; EC 2.6.1.7), KAT III (cysteine conjugate β-lyase 2, CCBL2; EC4.4.1.13) and KAT IV (mitochondrial aspartate aminotransferase, ASAT; EC 2.6.1.1). A previous study determined the relative contributions of KAT I, II, and IV to total KAT activity, and found that rat and human brain contain the highest proportion of KAT II (~60%) (with ~10 and 30% of KAT I and IV, respectively) suggesting a critical role for KAT II in KYNA synthesis in rat and human brain (Guidetti et al. 2007). KAT III contribution to brain KYNA synthesis remains to be determined. In the present study, glutamate, aspartate, cysteine, glutamine, and alanine suppressed KYNA production but not KYN uptake, suggesting that these amino acids inhibit the KYNA synthesis reaction. Glutamate and aspartate strongly inhibit KAT II (IC_50_: 2.1 and 1.2 mmol/L, respectively) and IV (IC_50_: 0.9 and 0.3 mmol/L, respectively), while glutamine and cysteine show inhibitory effects on KAT I and III activities (Guidetti et al. 2007; Han et al. 2009; Han et al. 2010). Furthermore, cysteine sulfinate, the deoxygenated product of cysteine, acts as a KAT II inhibitor and inhibits rat brain KYNA production at physiological concentrations *in vitro* (Kocki et al. 2003). Our results cannot determine if cysteine, cysteine sulfinate, or both inhibit the KYNA synthesis reaction. LAT 1 substrates (leucine, methionine, and phenylalanine) also inhibit KAT III activity (Han et al. 2009). In the present study, inhibition of KYNA production reflects reduced KYN uptake, and we did not observe additional effects of KAT inhibition.

We precisely investigated the effect of 10 amino acids on KYNA production and KYN uptake at 3 μmol/L–3 mmol/L. The physiological concentrations of leucine, isoleucine, phenylalanine, methionine, tyrosine, alanine, aspartate, cysteine, glutamine, and glutamate are approximately 150, 90, 60, 50, 70, 400, 10, 10, 700, and 80 μmol/L in rat plasma, respectively (Asai et al. 2008). Interestingly, all 10 amino acids partially inhibited KYNA production at physiological concentrations, with IC_50_ values of most amino acids for KYNA production or KYN uptake around physiological levels. Although the LATs mediating KYN uptake in blood–brain barrier is not completely identical to the tissue slices in the present study, it is expected that changes in physiological concentrations of these amino acids may affect brain KYNA levels *in vivo*. In particular, increases in plasma levels of these amino acids may lower brain KYNA levels.

Enhancement of brain KYNA production can be caused by pharmacological manipulation of KYN such as through systemic KYN administration or kynurenine 3-hydroxylase inhibitor *in vivo* (Swartz et al. 1990; Röver et al. 1997; Lombardi et al. 1994; Rassoulpour et al. 2005). Pharmacological manipulation of KAT suppresses KYNA production in the brain *in vivo* (Amori et al. 2009a; Amori et al. 2009b; Dounay et al. 2012; Kozak et al. 2014). In addition, recent studies have shown that diet also affects brain KYNA concentrations. High tryptophan diets increase brain KYNA levels owing to increased peripheral KYN in a dose-dependent manner, and reduce dopamine release via enhancement of KYNA production in the rat striatum (Okuno et al. 2011). Long-term exposure to a high-fat and low-protein/carbohydrate ketogenic diet shows a several-fold increase in KYNA concentrations in the rat brain (Żarnowski et al. 2012). Because amino acids are nutritional factors and often used as supplements, their side effects, safety doses, and interactions have been well investigated. Thus, long-term administration of amino acids from a diet may be a good method to manipulate KYNA formation in the brain. Several studies suggest that dietary large neutral amino acids modulated neurotransmitter release via LAT. For example, ingestion of α-lactalbumin-containing diet, a tryptophan-rich protein, increase brain Tryptophan content and serotonin synthesis and release (Choi et al. 2009; Orosco et al. 2004). Branched-chain amino acids ingestion causes the decline in tyrosine uptake and dopamine synthesis in brain (Fernstrom 2013). We suggest that large neutral amino acids may expand glutamate or acetylcholine release by suppressing KYNA production. In this study, we also show that non-large neutral amino acids, alanine, cysteine, glutamine and glutamate reduce KYNA production. Our findings may highlight the importance of dietary amino-acid compositions in brain chemistry and function. It will be interesting to determine the amino acid composition of habitual diets in patients with psychiatric disorders.

## Conclusions

We have investigated the effect of proteinogenic amino acids on KYNA production in rat brain *in vitro*. Our results show that amino acids can partially regulate KYNA production at physiological concentrations, and that some modulate KYN uptake, while others the KYNA synthesis reaction. Although animal studies are required to show the effect of these amino acids on KYNA production *in vivo*, amino acids have the potential to regulate KYNA formation and release of neurotransmitters such as dopamine, acetylcholine, and glutamate. Recent studies suggest that KYNA is involved in the pathophysiology of psychiatric disorders, including schizophrenia (Erhardt et al. 2007; Erhardt 2009). In addition, recent studies show that manipulations of peripheral KYN modulate depression-like behavior induced by stress (Agudelo 2014). Because diet affects brain KYN and KYNA concentration, long-term administration of amino acids through a diet may also affect KYNA production via regulating brain KYN uptake or KYNA synthesis. This approach may be useful in the treatment and prevention of neurological and psychiatric diseases associated with increased KYNA levels.

## Abbreviations

KAT: Kynurenine aminotransferase
KRB: Krebs-Ringer buffer
KYN: Kynurenine
KYNA: Kynurenic acid
LAT: Large neutral amino acid transporter
